# Modeling the role of incarceration in HCV transmission and prevention amongst people who inject drugs in rural Kentucky

**DOI:** 10.1016/j.drugpo.2020.102707

**Published:** 2020-03-06

**Authors:** Jack Stone, Hannah Fraser, April M Young, Jennifer R. Havens, Peter Vickerman

**Affiliations:** a Population Health Sciences, Bristol Medical School, University of Bristol, Oakfield House, Oakfield Grove, Bristol BS8 2BN, UK; b Department of Epidemiology, University of Kentucky College of Public Health, Lexington, Kentucky, USA; c Center on Drug and Alcohol Research, Department of Behavioral Science, University of Kentucky College of Medicine, Lexington, Kentucky, USA

**Keywords:** Hepatitis C virus, People who inject drugs, Incarceration, Prison, Mathematical modeling, Harm reduction

## Abstract

**Background:**

People who inject drugs (PWID) experience high incarceration rates, with current/recent incarceration being associated with increased hepatitis C virus (HCV) transmission. We assess the contribution of incarceration to HCV transmission amongst PWID in Perry County (PC), Kentucky, USA, and the impact of scaling-up community and in-prison opioid substitution therapy (OST), including the potential for reducing incarceration.

**Methods:**

A dynamic model of incarceration and HCV transmission amongst PWID was calibrated in a Bayesian framework to epidemiological and incarceration data from PC, incorporating an empirically estimated 2.8-fold (95%CI: 1.36–5.77) elevated HCV acquisition risk amongst currently incarcerated or recently released (<6 months) PWID compared to other PWID. We projected the percentage of new HCV infections that would be prevented among PWID over 2020–2030 if incarceration no longer elevated HCV transmission risk, if needle and syringe programmes (NSP) and OST are scaled-up, and/or if drug use was decriminalized (incarceration/re-incarceration rates are halved) with 50% of PWID that would have been imprisoned being diverted onto OST. We assume OST reduces reincarceration by 10–42%.

**Results:**

Over 2020–2030, removing the effect of incarceration on HCV transmission could prevent 42.7% (95% credibility interval: 15.0–67.4%) of new HCV infections amongst PWID. Conversely, scaling-up community OST and NSP to 50% coverage could prevent 28.5% (20.0–37.4%) of new infections, with this increasing to 32.7% (24.5–41.2%) if PWID are retained on OST upon incarceration, 36.4% (27.7–44.9%) if PWID initiate OST in prison, and 45.3% (35.9–54.1%) if PWID are retained on OST upon release. decriminalization (with diversion to OST) could further increase this impact, preventing 56.8% (45.3–64.5%) of new infections. The impact of these OST interventions decreases by 2.1–28.6% if OST does not reduce incarceration.

**Conclusion:**

Incarceration is likely to be an important contributor to HCV transmission amongst PWID in PC. Prison-based OST could be an important intervention for reducing this risk.

## Introduction

The U.S. has the largest global prison population (2121,600) (Walmsley, 2018) and one of the largest populations of people who inject drugs (PWID, estimated 2248,500) (Degenhardt et al., 2017). In the U.S., an estimated 3.4–6.0 million people have been exposed to HCV, with the HCV sero-prevalence in correctional facilities (~23.1% (Edlin, Eckhardt, Shu, Holmberg & Swan, 2015)) far exceeding that of the general population (1.7% (Edlin et al., 2015)). PWID also have a high HCV sero-prevalence in the U.S., with most new HCV infections occurring within this group (Edlin & Winkelstein, 2014). Recently, an epidemic of prescription opioid abuse in rural regions of the U.S. (Keyes, Cerda, Brady, Havens & Galea, 2014) led to an increase in the number of PWID (Peters et al., 2016) and an upsurge in reported cases of acute HCV infection (Suryaprasad et al., 2014).

In Perry County (PC), a rural county in Appalachian Kentucky, data from an on-going longitudinal cohort of PWID demonstrated a high sero-prevalence (58%) and incidence (19.4 per 100 person-years) of HCV among PWID. Furthermore, there is low coverage of opioid substitution therapy (OST, 4.7% coverage among community PWID (Havens et al., 2013)) and needle and syringe programmes (NSP) only initiated in 2018 (Kentucky Cabinet for Health & Family, Services).

Globally, PWID are frequently incarcerated (58% ever incarcerated (Degenhardt et al., 2017)), with data from Perry County (Young & Havens, 2012) suggesting that most PWID in that setting have ever been incarcerated (86%) and are incarcerated repeatedly (average 10 previous incarcerations) with short sentence lengths (average 3.4 months). Our recent systematic review suggests that recent incarceration could increase the risk of HCV acquisition among PWID by 62%, with data from PC suggesting a 2.8-fold increase in risk in that setting (Stone et al., 2018). Compounding this, there are no interventions provided for incarcerated PWID or targeted to recently released PWID in PC.

The high level of HCV transmission in PC, paired with high levels of incarceration and very low levels of interventions highlight an urgent need to develop strategies to reduce the transmission of HGV and levels of incarceration in this setting. This is crucial if there is to be any hope for rural US settings such as Perry County to reach the WHO goal of eliminating HCV as a public health threat by 2030 (Fraser et al., 2019). Prior modeling has generally only considered the need for community prevention and treatment interventions for reaching the WHO targets in PWID (Fraser et al., 2019; 2018; Gountas et al., 2017; Scott, McBryde, Thompson, Doyle & Hellard, 2017), with only our analysis from Scotland considering the role of prison-based interventions (Stone et al., 2017). In this analysis, we focus on evaluating the possible importance of both community OST (and NSP) and prison-based OST for helping PC achieve the WHO HCV elimination targets. We include direct benefits of OST on reducing HCV transmission risk (Platt et al., 2017), and the possible effect of OST on reducing incarceration rates based on data that it reduces criminal activity (Corsi, Lehman & Booth, 2009; Davstad, Stenbacka, Leifman & Romelsjo, 2009; Lawrinson et al., 2008; Sheerin, Green, Sellman, Adamson & Deering, 2004), criminal convictions (Bukten et al., 2012; Gossop, Trakada, Stewart & Witton, 2005; Keen, Rowse, Mathers, Campbell & Seivewright, 2000) and re-incarceration (Larney, Toson, Burns & Dolan, 2012; Werb et al., 2008).

## Methods

Throughout this paper, we use the term incarceration to refer to the act of detention of people in prisons, jails (the most common form of detention for the modelled population), or other closed settings and use the term prison to refer to any such setting where someone might be detained, including jail.

### Model description

**Fig. 1.**
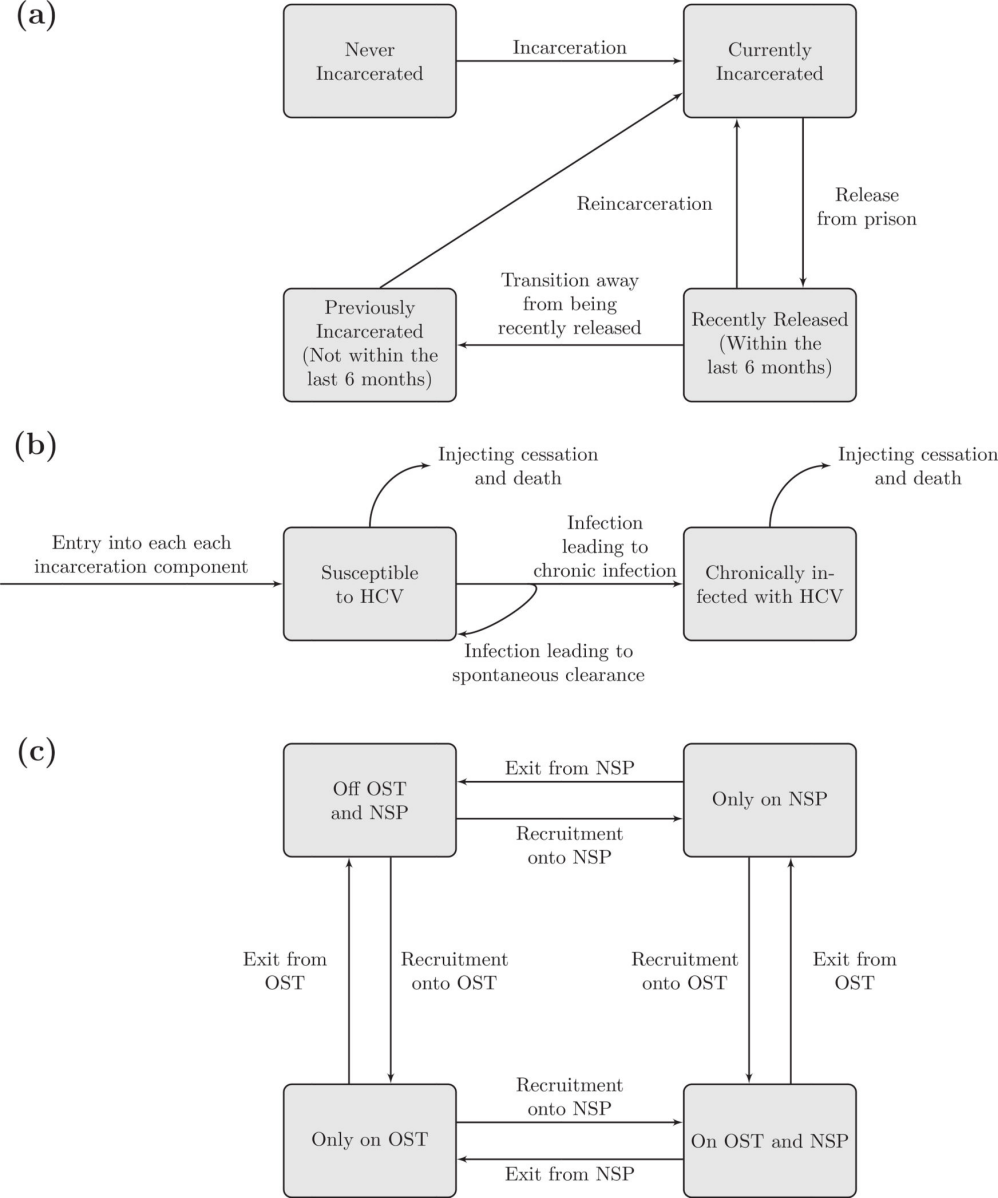
Model schematics of the incarceration component (a), HCV transmission component (b) and harm reduction states (c).

We adapted an existing dynamic, deterministic model of incarceration and HCV transmission amongst current PWID (Stone et al., 2017). The adapted model stratifies PWID by incarceration state (never incarcerated, currently incarcerated, recently released – last 6 months, previously incarcerated – released > 6 months ago) and HCV status (susceptible, chronically infected). The model additionally stratifies PWID by OST and NSP status (not on OST or NSP, on OST or NSP only, or on both OST and NSP; Fig. 1) in the community and prison. A full model description is in the supplementary materials.

All PWID enter the model as susceptible and not on OST or NSP, with a proportion entering each of the incarceration states. PWID are recruited onto and lost to follow-up from OST or NSP at constant rates which are independent of the other intervention state. In the status quo model, all PWID cease OST and NSP use upon incarceration, as currently occurs in PC. However, in some of the modelled intervention scenarios PWID are retained on OST upon incarceration. Community PWID experience reduced rates of incarceration whilst on OST (Larney et al., 2012). Depending on the intervention scenario modelled, PWID either leave OST or stay on OST upon release from prison.

PWID acquire and transmit HCV in their given setting (prison or community) with susceptible PWID becoming infected at a rate proportional to the chronic prevalence in their setting and the HCV transmission rate. The HCV transmission rate is reduced for PWID on OST and/or NSP and elevated if PWID are currently incarcerated or have been recently released (<6 months) from prison. The model assumes random mixing between all PWID risk subgroups within the community or prison.

### Model parameterisation and calibration

The model was parameterised and calibrated to the HCV epidemic among PWID in Perry County, Kentucky, with most parameter estimates coming from the Social Networks Among Appalachian People study (denoted as ‘SNAP’), the methods of which have been described previously (Young & Havens, 2012). The SNAP study recruited 503 illicit drug users (78% were initially PWID) through respondent driven sampling (RDS) in Perry County between November 2008-September 2010 and followed them up between every six and eighteen months until 2014/15.

Model parameterisation and calibration comprised two steps. First, the incarceration dynamics were parameterised and calibrated to self-reported incarceration history data. This was done with an adapted incarceration model (see supplementary materials) that simulated a cohort of PWID progressing through their injecting career with the sub-model (Supplementary Fig. S1) being calibrated to incarceration-related data. The HCV transmission component of the model was then parameterised and calibrated, using incarceration parameters from the first step and incorporating an increase in the initiation rate of new PWID between 1990 and 2000.

### Step 1: Parameterising and calibrating the incarceration sub-model

**Fig. 2.**
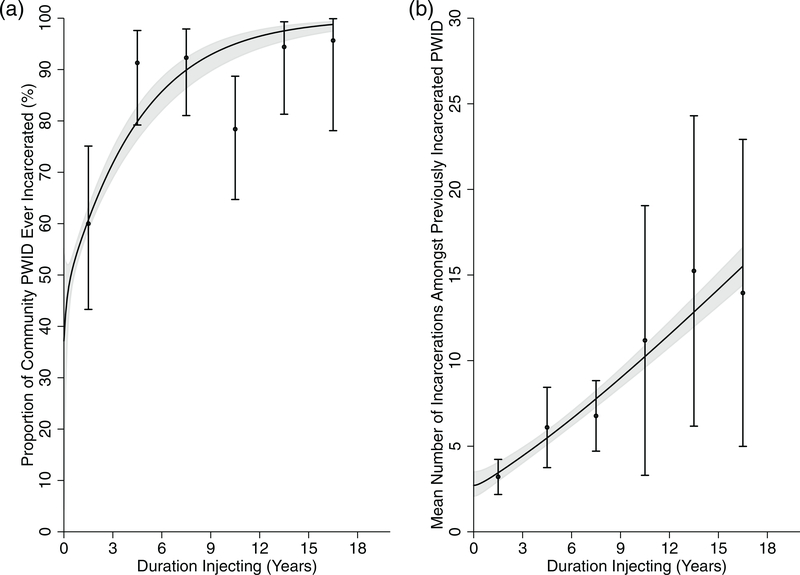
Model fits of the incarceration component of the model to (a) the proportion of community PWID previously incarcerated and (b) the mean number of incarcerations, by duration of injection. Lines represent the median of all fits, with the shaded area representing the 95% credibility interval of the fits. Data points estimated from SNAP (circles), with their 95% confidence intervals (whiskers), used in the fitting procedure are shown for comparison.

We tracked a simulated cohort of 1000 PWID for 18 years from initiation of injecting to calibrate the model's incarceration and re-incarceration rates, and proportion of new PWID initiating injecting in each incarceration state. An approximate Bayesian computation sequential Monte Carlo scheme (Toni, Welch, Strelkowa, Ipsen & Stumpf, 2009) was used to obtain a sample of 5000 incarceration-related parameter sets (prior and posterior parameter ranges in Table 1) that fit the SNAP incarceration data on the proportions of community PWID who have ever been incarcerated and the mean number of incarcerations by duration injecting (data shown in Fig. 2). Full details of this calibration process are in the Supplementary materials. Fig. 2 shows the model fits to data on the proportion of PWID ever incarcerated and their mean number of incarcerations by duration of injecting.

### Step 2: Parameterising and calibrating the full model

SNAP data suggest community OST coverage was 4.7% (95% CI: 3.8–5.8%) in 2009, which remained stable throughout the study, with SNAP data suggesting a mean duration of 5.6 months (95%CI: 3.3–9.1 months) on OST. There was negligible NSP. Two studies from Australia and Canada suggest OST reduces re-incarceration rates by 20% (AHR: 0.80, 95% CI: 0.71–0.90) (Larney et al., 2012) or 34% (AOR: 0.66, 95% CI: 0.58–0.76) (Werb et al., 2008), respectively, with other studies suggesting similar reductions in the incidence of criminal convictions (Bukten et al., 2012; Gossop et al., 2005; Keen et al., 2000). We, therefore, assume that OST reduces incarceration by 10–42% (range from confidence intervals for two estimates above). We assume PWID inject for 5–25 years.

For each of the 5000 incarceration-related parameter sets obtained from step 1, parameter values for the transmission component of the model were sampled from their probability distributions (Table 2). For each of these 5000 full parameter sets, the time-varying number of individuals initiating injecting annually and their cessation rate were calibrated (using nonlinear least-squares methods) to give the estimated PWID population size for Perry County in 2009 (700+/−20%, analysis for this project), while assuming up to an 8-fold increase in the number of individuals initiating injecting over 1990–2000, consistent with data (Zibbell et al., 2015) (see supplementary materials), with initiation of injecting assumed to be constant after 2000. OST recruitment and loss to follow-up rates were then calibrated (using nonlinear least-squares methods) to sampled values of OST coverage level and average duration, while assuming that discontinuation of OST upon incarceration also contributes to loss to follow-up. Finally, the community HCV transmission rate and increased risk for currently/recently incarcerated PWID was calibrated (using nonlinear least-squares methods) to fit the model to the sampled antibody HCV prevalence (52.2–63.6%) and IRR (1.36–5.77), assuming that the HCV epidemic was stable prior to 1990 when the initiation rate of PWID increased. Model fits were accepted, and subsequently used in the model analyses, if the projected HCV incidence among all PWID for 2011 was within the 95% confidence intervals for the estimated HCV incidence (14.9–23.7 per 100py) from the SNAP cohort for 2009–2014.

## Model analyses

### Contribution of incarceration to HCV transmission amongst PWID

The calibrated model was firstly used to project the contribution or “population attributable fraction” (PAF) of incarceration to current HCV transmission amongst PWID in Perry County. This was done by comparing the number of incident infections that occurred from 2020–2030 in the status quo scenario, which included an elevated risk of HCV transmission amongst currently incarcerated PWID and recently released PWID, with how many that would occur if incarceration had no effect on transmission risk from 2020–2030. This was modelled by removing the excess HCV transmission risk amongst currently incarcerated and recently released PWID from 2020 onwards. Both scenarios assumed current low levels of OST and no NSP. The projected relative reduction in incident infections over 10 years was defined as the PAF of incarceration.

### Impact of scaling-up OST and NSP and reducing incarceration rates amongst PWID

The model was then used to project the 10-year impact (2020–2030) of several intervention scenarios (S1-S4) which included scaling-up community harm reduction, various aspects of prison-based OST, and/or reducing incarceration rates for PWID to simulate what may occur with decriminalization (S5-S7). For scenarios where we assume some coverage of NSP, an average duration on NSP of 9.1–10.7 months was used based on data from a three-city study in the U.S. (Green et al., 2010) (see supplementary materials). Prison NSP was not considered. Specifically, modelled scenarios are as follows:

**Status quo Scenario:** Community OST coverage remains low with no NSP.

Scenario S1*: Scale-up community OST to 50% coverage amongst community PWID, but no retention upon incarceration.

**Scenario S1:** Scale-up community OST and NSP to 50% coverage amongst community PWID, but no retention upon incarceration.

Scenarios S2/S2*: Scenario S1/S1* plus retain PWID on OST upon incarceration. Prison OST is assumed to have the same retention rate as community OST, but all leave OST on release.

Scenarios S3/S3*: Scenario S2/S2* plus recruit incarcerated PWID onto OST at the same rate as community PWID.

Scenarios S4/S4*: Scenario S3/S4* plus retain all PWID on OST for 6 months following release, defined as ‘Comprehensive harm reduction’.

**Scenario S5:** Scenario S4 plus assume drug use is decriminalized. We model decriminalization similar to the Portuguese model such that PWID would not be incarcerated for drug possession-based charges but instead would be referred to voluntary treatment. As an illustrative example, due to a lack of data on offences leading to incarceration among PWID in Kentucky, we assume half of all incarceration events are due to possession and that halve of those referred to OST would start treatment. Specifically, we assume that (re-)incarceration rates are reduced by half, and 50% of PWID that would have been incarcerated are diverted onto OST (i.e. 50% are incarcerated, 25% remain in the community off OST, 25% remain in the community but are diverted onto OST).

The impacts of these scenarios were estimated by comparing the percentage of incident HCV infections averted over the 10-year period 2020–2030. The model was also used to consider how the projected impacts would differ if there was no effect of OST on rates of incarceration and re-incarceration.

### Uncertainty analysis

A linear regression analysis of covariance (ANCOVA) was undertaken to determine which parameter uncertainties contribute most to uncertainty in the model projections of the impact of the most comprehensive community and prison harm reduction (S4). The proportion of each model outcome's sum-of-squares contributed by each parameter was calculated to estimate the importance of individual parameters to the overall uncertainty.

## Results

### Status quo model projections

The model suggests that 21.9% (95% credible interval (CrI): 17.9–27.6%) of PWID are currently incarcerated in 2019, with an initial incarceration rate of 23.8% (19.3–27.8%) per year and a subsequent re-incarceration rate of 127.0% (109.5–144.5%) per year, i.e. average time to re-incarceration of 9.4 months. The model suggests that on average PWID inject for 8.3 years (5.2–12.9 years), during which time they are incarcerated 6.8 times (4.0–11.2). Fig. 2 shows the fit of the incarceration sub-model to available incarceration data from the SNAP cohort. Overall, the model also suggests that PWID are incarcerated for 22.2% (18.2–28.1%) of their injecting career (1.8 years; 1.0–3.2 years) and are in the post-release higher transmission risk period for 24.9% (22.8–26.8%) of their time.

**Fig. 3.**
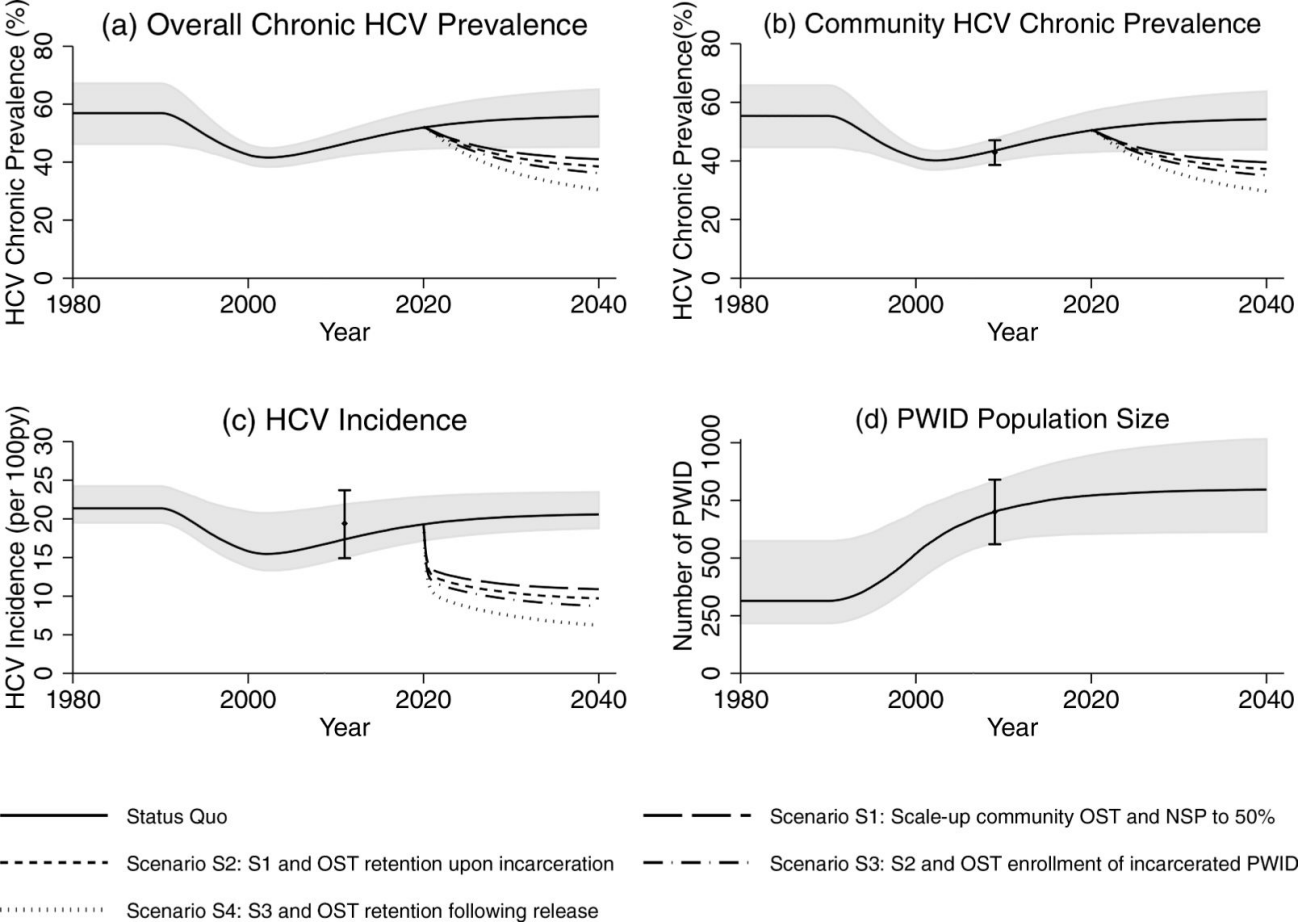
Model projections of (a) overall chronic HCV prevalence among all PWID (in the community and in prison), (b) chronic HCV prevalence among community PWID, (c) HCV incidence, and (d) PWID populations size. Lines represent the median HCV chronic prevalence, HCV incidence and PWID population size. The shaded area represents the 95% credibility intervals for status quo projections. HCV incidence and prevalence data points are shown for comparison with 95% confidence intervals. OST denotes Opioid substitution therapy and NSP denotes Needle and syringe programmes.

The status quo projections suggest that the PWID population has increased by 47.7% (29.3–141.4%) over 1990–2020 (Fig. 3), from 314.1 (217.5–573.9) to 771.3 (605.1–946.0). As a consequence of the increase in initiation of injecting, the model projects that HCV chronic prevalence and incidence declined by about 40% over 1990–2000, to 41.6% (38.4–44.8%) and 15.5 (13.3–20.7) per 100py in 2002, respectively. HCV chronic prevalence and incidence then increased to 51.9% (44.6–58.2%) and 19.3 (17.2–22.9) per 100py in 2020, and will increase further by 2030 (Fig. 3).

### Contribution of incarceration to HCV transmission amongst PWID

The model projects that over 2020–2030, removing the effect of incarceration (and recent release) on elevating HCV transmission would prevent 42.7% (15.0–67.4%) of new HCV infections, reducing the number of new HCV infections in PC from 723.0 (550.9–949.6) to 413.7 (224.8–673.7). Hence, incarceration of PWID contributes two-fifths of on-going HCV transmission amongst PWID or the PAF of incarceration is 42.7%. Importantly, removing the effect of incarceration reduces HCV incidence by 57.5% (20.7–81.5) by 2030, with HCV incidence decreasing to 8.2 (3.6–15.5) per 100py.

### Impact of scaling-up harm reduction

**Fig. 4.**
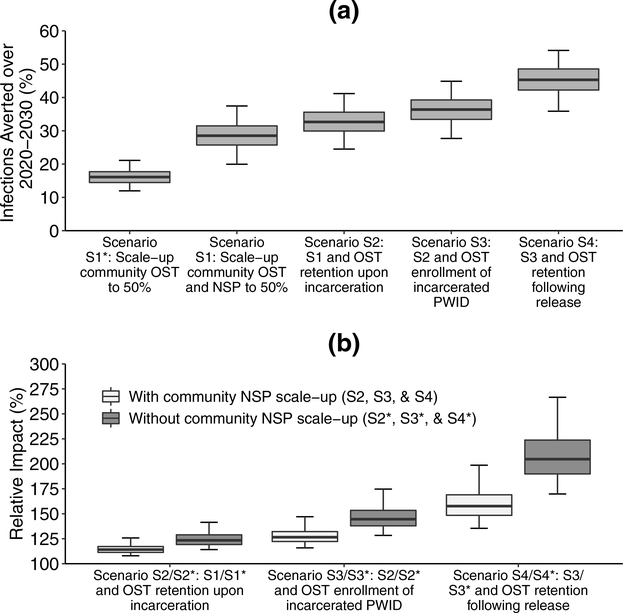
Impact of scaling-up Opioid substitution therapy (OST) and Needle and syringe programmes (NSP) for various scenarios with differing levels of OST scale-up in prisons. Fig. 4a shows the percentage of incident HCV infections that would be averted over 10 years compared to the status quo scenario, while Fig. 4b shows the relative impact compared to scaling-up OST only in the community with (green – Scenario S1) and without (red – Scenario S1*) a concurrent NSP scale-up among community PWID. Bars show the median projections, boxes show the interquartile range, while error bars show the 95% credibility intervals.

**Fig. 5.**
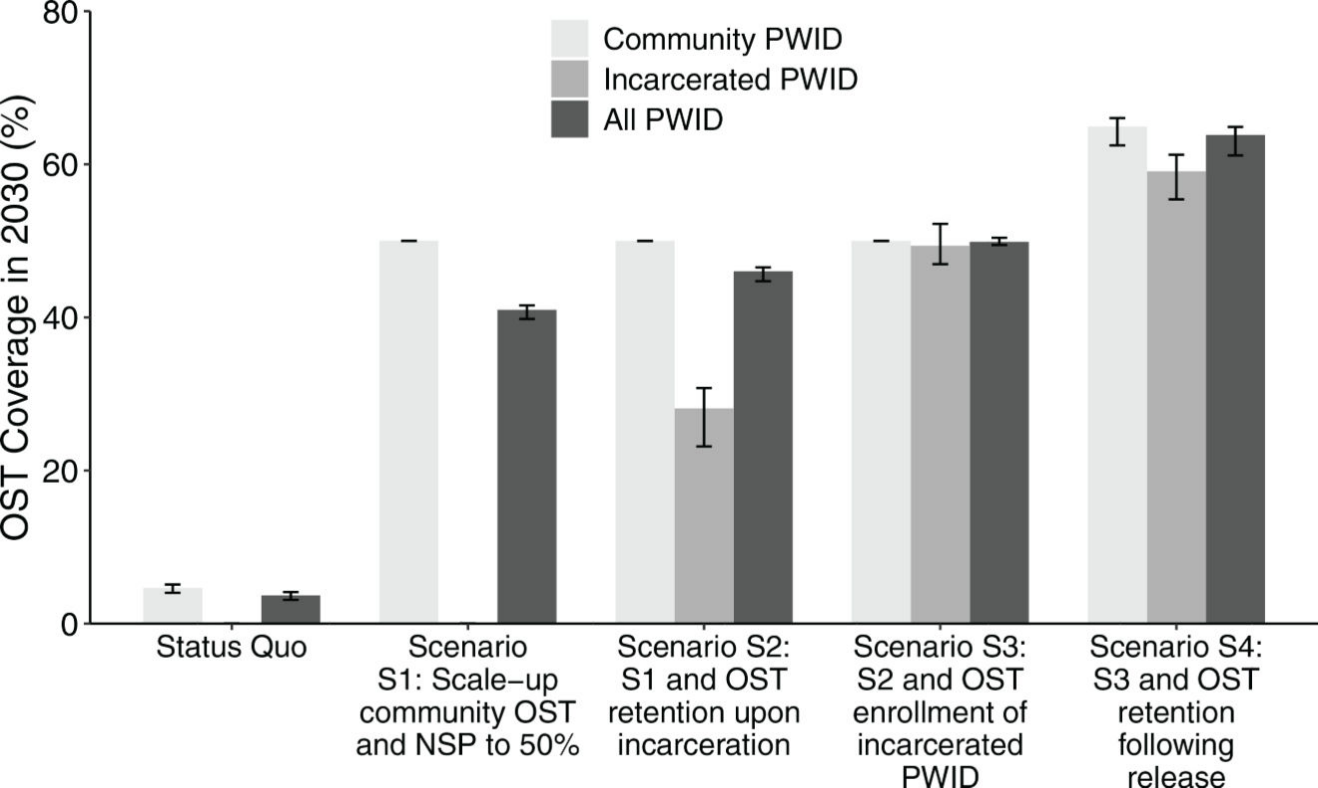
OST coverage among community PWID (light gray), incarcerated PWID (medium gray) and all PWID (dark gray) for each modelled intervention scenario with differing levels of OST scale-up in prisons. Bars show the median projections, while error bars show the 95% credibility intervals.

Scaling-up OST and NSP to 50% coverage amongst community PWID (S1) could avert 28.5% (20.0–37.4%) of incident HCV infections over 2020–2030 (Fig. 4) reducing HCV incidence by 40.4% (28.0–53.5%) to 11.5 (9.2–14.3) per 100py. This impact increases to 32.7% (24.5–41.2%) of HCV infections being averted if PWID are retained on OST when incarcerated (S2), increasing to 36.4% (27.7–44.9%) if incarcerated PWID are also recruited onto OST in prison (S3), and 45.3% (35.9–54.1%) if PWID are also retained on OST for 6 months upon release from prison/jail (“comprehensive harm reduction”; S4). This results in a relative 60.0% (43.7–87.8%) greater overall coverage of OST (Fig. 5) and 57.7% (35.4–98.6%) greater impact than was achieved from just scaling-up OST and NSP amongst community PWID (Fig. 4). In the comprehensive harm reduction scenario S4, HCV incidence would reduce by 61.3% (49.6–72.4) by 2030 (to 7.5 (5.6–9.8) per 100py) compared to the status quo projections (Fig. 3). Importantly, compared to a scenario where just community OST is scaled (S1*), the added components of prison OST in scenario S4* result in a doubling in impact (104.6% (69.8–166.6%) increase) in terms of infections averted (Fig. 4).

If OST has no effect on incarceration rates from 2020 onwards, then reduced coverage levels of OST and NSP would occur among community PWID, 47.4% (44.7–49.1%) and 48.2% (47.1–49.3%), respectively, instead of 50% amongst community PWID for scenario S1 in 2030, with 14.9% (5.7–28.6%) less infections being averted. This effect reduces for the scenarios where incarcerated PWID also access OST, with 5.9% (2.1–13.0) less infections averted for scenario S4. In other words, if OST reduces incarceration, then the percentage of HCV infections averted due to OST in the community increases relatively by 2.1–14.9% or 6.0–40.1% depending on whether OST is scaled-up in prisons or not, respectively.

### Impact of decriminalization

When in addition to comprehensive harm reduction (S4), we also assume decriminalization occurs from 2020, then 56.8% (45.3–64.5%) of incident HCV infections over 2020–2030 could be prevented if incarceration rates for PWID are halved and 50% of these PWID are diverted to OST (S5). This is partly due to the coverage of NSP increasing because it is not being disrupted by periods of incarceration. Interestingly, similar impact is achieved for this scenario if there was no diversion to OST because OST coverage levels are already high. Conversely, diverting PWID to OST from prison is more important if community interventions are limited as they currently are. In this scenario, diverting 50% of to-be incarcerated PWID to OST could prevent 15.2% (7.0–22.3) of incident HCV infections over 2020–2030, compared to 12.1% (3.3–19.7) if decriminalization just halved incarceration rates.

### Uncertainty analysis

Analysis of covariance indicated that uncertainty in the reduced risk of acquiring HCV whilst on OST (42.3%) or NSP (41.7%), the rate of mortality and cessation (7.2%) and the OST loss to follow-up rate (4.2%) contributed most to the variability in the impact of scaling-up OST and NSP to comprehensive harm reduction (S4). No other model parameters contributed more than 2% to the variability (Supplementary Fig. S4).

## Discussion

### Main findings

Due to frequent incarceration and elevated acquisition risk associated with incarceration and recent release, our modeling suggests incarceration could be contributing two-fifths (42.7%) of ongoing HCV transmission amongst PWID in Perry County. If this elevated risk could be prevented, HCV incidence could be reduced by 57.5% by 2030. Because of negligible coverage of OST and NSP in this setting, scaling-up these interventions to 50% coverage among community PWID could also avert a quarter (28.5%) of incident HCV infections over 2020–2030. However, the impact of these interventions is hindered by the frequent incarceration of PWID. Indeed, if PWID were also retained and recruited onto OST while incarcerated and retained on OST following release then nearly half (45.3%) of all incident HCV infections could be prevented. decriminalization could also be important, with a halving in incarceration rates increasing impact with 56.8% of infections being prevented. The diversion of to-be incarcerated PWID to OST could also be an important strategy but only if OST provision in prisons is low.

### Strengths and limitations

This study presents the first analysis of the impact of HCV prevention interventions for both community and incarcerated PWID in a U.S. setting, and the first to estimate the contribution of incarceration to HCV transmission in a U.S setting. The main strength of this study is the use of detailed data from Perry County to inform model parameterisation, including context specific data on mortality and OST retention. Despite this, the study still had limitations.

Firstly, the findings may not be generalisable to urban U.S. or other high-income country settings as the model was parameterised to a rural county in Appalachian Kentucky, which has high rates of incarceration (86% PWID ever incarcerated) and low intervention coverage. Many cities in the U.S. and other high-income countries have much higher levels of intervention coverage (Fraser et al., 2019; Larney et al., 2017) and some already have OST in prison (Zurhold & Stöver, 2016); less impact would be achieved from scaling-up interventions in these settings. Furthermore, the model simulates an increasing injecting population and HCV epidemic, which although consistent with the growing epidemic of prescription opioid abuse in the U.S., is not applicable to many other settings (Lindenburg et al., 2006). There is also likely to be other variability across U.S. settings which may affect the generalisability of these results, particularly other differences between urban and rural settings. This could include duration of injecting, which is likely to be less in rural settings than urban settings, and lower levels of substance abuse treatment in rural areas (Meit et al., 2014), both of which could affect the impact of scaling-up prevention interventions (Martin, Hickman, Hutchinson, Goldberg & Vickerman, 2013).

Secondly, uncertainty exists over the magnitude and duration of the heightened risk related to incarceration. Whilst we used an empirical estimate from a cohort in Perry County, only those that reported incarceration in the last 30 days before the interview, which were on average 6 months apart, were classified as exposed to incarceration in our analyses. This will have resulted in some incarceration events being missed and allocated incorrectly because they occurred over 30 days before the interview, likely resulting in us under-estimating the elevated HCV transmission risk related to incarceration and so the contribution of incarceration to HCV transmission in this setting. Furthermore, we assumed the same increase in risk during periods of incarceration and the 6-month period following release, as we were unable to separate the risk associated with these two periods in the cohort. If, however, the risk in prison is lower than the period following release, then the risk following release would need to be higher to reproduce the observed heightened risk of HCV acquisition associated with recent incarceration, giving a larger impact of OST after release than modelled but a similar contribution of incarceration to HCV transmission.

Thirdly, available data from the SNAP cohort could not distinguish between detention in prison or jail, although most incarceration events within this cohort are thought to be within jails. The inability to distinguish between these types of incarceration and the possible undersampling of those who have longer times in incarceration may have led to an underestimation of the average duration of detention, and so the impact of prison-based OST. Furthermore, it is possible that the increase in risk following release from prison or jail may differ, although data was lacking. However, evidence from Montreal suggests that the effect of recent incarceration is similar by incarceration type (jail or prison) (Artenie, 2019).

Fourthly, although much of the data used to parameterise the model is specific to Perry County, some were acquired from elsewhere. For instance, rates of leaving NSP were unavailable for this setting due to there currently being negligible NSP in Perry county. The NSP exit rate, therefore, was parameterised using data from other U.S. sites (Green et al., 2010). It is likely that the average duration in contact with NSP will differ based on the characteristics of the NSP initiated in Perry County, including opening times, location, and the range of services provided. Future analyses should consider locally specific data.

Fifthly, baseline model fits assume no NSP. In practice, an NSP has been operating in Perry County since 2018 with limited opening hours; 4 hours a week at first which has now been expanded to 9am to 2pm, four days a week. Based on the average frequency of injecting (estimated as 26–44 times per month based on SNAP cohort data) and the number of needles distributed since 2018 (96,328), we estimate that 62 syringes have been distributed per PWID per year in Perry County. At best, this means that 91–154 (12–20%ofPWIDinPerryCounty)PWID could obtain enough needles to ensure all (100%) their injections are safe. This is a low coverage that would have a small impact on HCV transmission (Platt et al., 2017). Therefore, given the recency of the NSP and its low coverage, our baseline model assumed no NSP. Our first intervention scenario incorporates a scale-up in NSP coverage to 50% from 2020, demonstrating the impact that further NSP expansion will have in this setting.

Finally, whilst it is widely accepted that OST is effective at reducing criminal activity (Davstad et al., 2009; Lawrinson et al., 2008; Mattick, Breen, Kimber & Davoli, 2009; Sheerin et al., 2004), the extent to which this translates to a reduction in incarceration has only been estimated in two studies (Larney et al., 2012; Werb et al., 2008). Since the effect of OST on reducing incarceration differed widely between these studies, we considered a wide range in our analysis. As the effect of OST on incarceration rates may depend on numerous factors, including the proportion of incarcerations that are due to drug-related crime, future analyses should consider context-specific estimates for this parameter.

### Comparisons with existing studies

This analysis is consistent with previous modeling of the contribution of incarceration to HCV and HIV transmission in Scotland (Stone et al., 2017) and Ukraine ( Alticeet al., 2016). As with these prior studies, this work finds that incarceration is likely to be an important contributor to HCV transmission amongst PWID. Previous modeling has considered the cost-effectiveness of prison-based HCV treatment in the US (Assoumou et al., 2019; He et al., 2016), with only one study considering the impact on HCV transmission (He et al., 2016). Our modeling is consistent with that modeling (He et al., 2016) and others (Stone et al., 2017) in showing that prison-based prevention and treatment interventions can reduce HCV transmission in the community. This work is also consistent with previous modeling studies evaluating the impact of OST and NSP in the community (Vickerman, Martin, Turner & Hickman, 2012). This analysis adds to these by showing that if OST is not provided for incarcerated PWID, as is currently the case in the U.S., the impact of scaling-up community OST and NSP will be limited by high levels of incarceration, with this being compounded by elevated HCV transmission risk during incarceration and following release. This agrees with previous modeling of HIV in Ukraine (Altice et al., 2016) that showed the important prevention benefit of providing OST for incarcerated PWID and ensuring retention following release. Indeed, our study highlights that retaining PWID on OST upon incarceration, enrolling incarcerated PWID on OST and retaining them upon release can achieve much greater (62% more) impact than just scaling-up OST and NSP in the community. This is consistent with our recent modeling that highlighted the importance of scaling-up OST in prisons alongside the community for reducing mortality among PWID in Kentucky and other settings (Degenhardt et al., 2019). Lastly, this is the first modeling study to quantify the additional prevention benefits that may occur due to PWID on OST having reduced rates of incarceration, which suggests it could increase the impact of OST by up to 40.1%.

### Implications

The findings add to a growing body of evidence that suggests incarceration is an important contributor to HCV transmission amongst PWID, adding new evidence for a U.S. setting with very high levels of incarceration. In contrast to many settings, community coverage levels of OST are very low and NSP has only just been initiated in Perry County, so the detrimental effect of incarceration in Perry County is unlikely to be due to reduced coverage of harm reduction interventions among incarcerated or recently incarcerated PWID. Similarly, few PWID (5.6% in past 3 years) experience homelessness in Perry County (Young & Havens, 2012), and so it is unlikely that homelessness plays a role in elevating HCV transmission following prison release. For developing future interventions, it is important to investigate the possible mechanisms by which incarceration elevates HCV transmission in Perry County. This is crucial for intervention development and for advocating for decriminalization, which our analysis suggests could be a powerful public health strategy in the U.S.

To our knowledge, this is the first study to assess the possible impact on HCV transmission of OST reducing incarceration. Our findings suggest important prevention benefits (up to 40.1% more HCV infections averted) could result from this effect, although our projections are uncertain. It is important that further studies evaluate this effect of OST on incarceration so that its additional prevention benefits can be quantified better. This is also important because previous economic analyses have shown that reductions in crime and incarceration are crucial for determining the cost-effectiveness of OST (Connock et al., 2007; Kenworthy et al., 2017).

International evidence has shown that prison-based OST is associated with reductions in injecting risk (Hedrich et al., 2012), can significantly reduce HCV incidence amongst incarcerated PWID, as suggested by the 5-times lower HCV incidence among PWID on MMT compared to PWID not on MMT in a Spanish prison (Marco, Gallego & Cayla, 2014) and the approximate three-quarter reduction in HCV incidence after the introduction of OST in Scottish prisons (Champion, 2004; Marco et al., 2014; Taylor et al., 2013), and reduces mortality amongst opioid dependent prisoners; both during incarceration (Larney et al., 2014) and following release ( Degenhardtet al., 2014; Marsden et al., 2017), a period that is otherwise associated with elevated risk of drug-related deaths (Merrall et al., 2010). However, OST is abruptly stopped upon incarceration in most U.S. jurisdictions (Rich et al., 2015), with exceptions sometimes made for pregnant women (Fiscella, Moore, Engerman & Meldrum, 2004) or people with HIV (Rich et al., 2015). In contrast, in many Western European countries, OST is continued and also initiated in prison (Zurhold & Stöver, 2016). Our findings suggest the continuation of OST upon incarceration, recruitment onto OST in prisons and retention on OST following release are crucial for maximizing the impact of OST on HCV transmission. Without such a comprehensive approach to harm reduction, periods of high transmission risk associated with current and recent incarceration will persist, which could undermine future efforts to achieve HCV elimination through scaled-up harm reduction and HCV treatment. A comprehensive approach to harm reduction, which includes prison-based OST, could be a strong platform for achieving HCV elimination, and would optimize the impact of HCV treatment.

## Supplementary Material

1

## Figures and Tables

**Table 1:** Prior and posterior model parameter ranges for the incarceration sub-model.

Parameter	Prior Distribution	Posterior parameter range	Source/notes
Average time in prison per incarceration (mths)	Normal 3.4 (95% CI: 2.7–4.1) truncated to 95% confidence interval.	2.7–4.1	SNAP cohort
Percentage of PWID initiating injecting when			Prior for p1 is from SNAP data analysis.
Never incarcerated (p1)	Uniform [43.5,62.7%]	43.5–50.8	Uninformative prior for p2.
Incarcerated (p2)	Uniform prior [0,1-p1]	0.0–56.1	
Previously incarcerated (p3)	p3 = 1-p1-p2	0.0–56.1	
Incarceration rate per year	Uniform [0,1]	0.17–0.29	Uninformative prior
Re-incarceration rate per year	Uniform [0,2]	1.0–1.5	Uninformative prior
Mean number of incarcerations amongst PWID initiating injecting	Uniform [1,7]	2.0–3.8	Uninformative prior. Used in model calibration only – not in final model.

**Table 2:** Full model parameters obtained from literature and data analyses.

Parameter	Range of parameter values	Source/Notes
PWID and HCV-related parameters		
Anti-HCV prevalence amongst community PWID	58.0% (95%CI: 52.2–63.6)	SNAP cohort. Sampled from normal distribution truncated to 95% CI.
Average proportion of infections that spontaneously clear	0.26 (95%CI: 0.22–0.29)	(Micallef, Kaldor & Dore, 2006) Sampled from uniform distribution.
Rate at which PWID initiate injecting (Per year)	Varied over time	Calibrated to PWID population size in 2009.
Factor increase in initiation rate of injecting drug use in 1990–2000	1.02–6.32	Calibrated so that among current PWID in 2009 there would be 8 times more PWID that started injecting in 2000 than in 1990.
PWID population size in 2009	560–840	SNAP data analysis estimates a PWID population of 700. Sampled from uniform distribution.
Mortality rate (Per 10,000 person years)	50–130	SNAP data analysis. Sampled from Poisson distribution with rate 88.
Average duration of injecting in years	5–25	Young expanding population of injectors so uncertainty in duration of injecting - wide range assumed with uniform distribution.
Rate ratio for acquiring HCV if currently or recently incarcerated.	2.80 (95%CI: 1.36–5.77)	(Stone et al., 2018) Sampled from lognormal distribution, truncated to 95% CI.
**Harm Reduction parameters**		
Community OST loss to follow-up rate (per year)	0.44–2.90	SNAP data analysis gives overall OST exit rate 1.3–3.5 per year. Community OST loss to follow-up rate is calibrated to give this OST exit rate, sampled from uniform distribution
NSP loss to follow-up rate (per year)	1.12–1.32	Estimated from Green et al. (2010). See supplementary materials.
OST recruitment rate (Per Year)	Model calibrated	Varied to give coverage of 4.7% (95% CI: 3.8–5.8%) in 2009 (SNAP data, normal distribution) and then increased from 2020 to give different OST coverage scenarios.
OST start date	1990–1999	Sampled from uniform distribution
Relative incarceration rates while on OST.	0.58–0.90	Sampled from uniform distribution. Details in main text.
NSP recruitment rate (per Year)	Model calibrated	No NSP in status quo. Varied to give required coverage for intervention.
Relative risk of acquiring HCV while on:		(Platt et al., 2017) Sampled from lognormal distribution, truncated to 95% CI. Efficacy while on OST and NSP assumed to be product of individual effects. Efficacy of OST in prison is assumed to be the same as in the community.
OST only	0.50 (95% CI: 0.40–0.63)	
NSP only	0.44 (95% CI: 0.24–0.80)	
