# Supplementary material for: Modeling the role of incarceration in HCV transmission and prevention amongst people who inject drugs in rural Kentucky
Source: Int J Drug Policy. Author manuscript; Available in PMC 2021 Mar 29. (PMC7483428; doi:10.1016/j.drugpo.2020.102707)
Supplement: 1 [file NIHMS1607793-supplement-1.docx]

**Supplementary Materials to Modelling the Role of Incarceration in HCV Transmission and Prevention Amongst People Who Inject Drugs in Rural Kentucky**

Table of Contents

[Model equations 2](#_Toc22197633)

[Parameterising and calibrating the incarceration sub-model 6](#_Toc22197634)

[Parameterising and calibrating the full model 8](#_Toc22197635)

[Calibrating the increase in injecting initiation 8](#_Toc22197636)

[Parameterising the effect of OST on incarceration rates 10](#_Toc22197637)

[Parameterising NSP loss to follow-up rates 10](#_Toc22197638)

[Uncertainty Analysis 12](#_Toc22197639)

## Model equations

The model includes compartments for susceptible PWID ${(X}_{i,j})$ and chronically infected PWID ${(C}_{i,j})$. The model population was stratified by incarceration status: never incarcerated, currently incarcerated, recently released (within the last 6 months) and previously incarcerated but not in last 6 months ($i=0, 1, 2, 3$ respectively). The model population was further stratified by OST and NSP status: not on OST or NSP, on OST only, on NSP only or on both OST and NSP (j=0, 1, 2 ,3 respectively).

New PWID enter the model at rate $\theta(t)$ as uninfected PWID not on OST or NSP, with a fixed proportion $p_{i}$ of entrants entering each incarceration state. PWID leave all compartments through death or permanent cessation of injecting with rate $\mu$.

PWID in the community are incarcerated with rates dependent upon their incarceration state, with never incarcerated PWID experiencing incarceration with rate $\gamma$ and recently released and previously incarcerated PWID experiencing a re-incarceration rate $\delta$. PWID receiving OST experience a factor $\psi$ lower rate of incarceration and re-incarceration. Incarcerated PWID are released from prison with rate $\tau$ after which PWID spend an average of 6 months in the recently released compartment before transitioning to the previously incarcerated compartment.

PWID are recruited onto OST and NSP at rates $\omega_{i}(t)$ and $\xi_{i}\left( t \right)$, respectively, which are both time dependent and depend on PWID’s incarceration state. PWID leave OST and NSP at rates, $\zeta$ and $\pi$. A proportion, $c$, of PWID are retained on OST upon incarceration and a proportion, d, of PWID are retained on OST upon release from prison/jail. All PWID leave NSP upon incarceration.

All PWID are initially susceptible ${(X}_{i,j})$ and become HCV infected with a per-capita rate, λ specific to their incarceration status. Forces of infection are given by$\beta^{p}$ for incarcerated PWID and$\beta^{c}$ for community PWID,). The force of infection is heightened by a factor $\eta$ amongst currently incarcerated and recently released PWID but lowered by factors $\phi, \chi$ and $\phi\chi$ for PWID on OST only, NSP only or both OST and NSP, respectively. Incarcerated PWID can only transmit HCV to other incarcerated PWID and similarly community PWID can only transmit HCV to other PWID in the community. A proportion ($\alpha$) of PWID spontaneously clear their acute infection and remain susceptible ${(X}_{i,j})$, with the remaining proportion $(1-\alpha)$ proceeding to the chronically infected compartment ${(C}_{i,j})$.

The full model equations are as follows, for PWID who have never been incarcerated:

$$\frac{\partial X_{0,0}}{\partial t}=p_{o}\theta(t)-\left( \mu+\gamma+\omega_{0}\left( t \right)+\xi_{0}\left( t \right)+\left( 1-\alpha\right)\beta^{c} \right)X_{0,0}+\zeta X_{0,1}+\pi X_{0,2}$$

$$\frac{\partial C_{0,0}}{\partial t}=\left( 1-\alpha\right)\beta^{c}X_{0,0}-\left( \mu+\gamma+\omega_{0}\left( t \right)+\xi_{0}\left( t \right) \right)C_{0,0}+\zeta C_{0,1}+\pi C_{0,2}$$

$$\frac{\partial X_{0,1}}{\partial t}=-\left( \mu+\psi\gamma+\zeta+\xi_{0}\left( t \right)+\phi\left( 1-\alpha\right)\beta^{c} \right)X_{0,1}+\omega_{i}\left( t \right)X_{0,0}+\pi X_{0,3}$$

$$\frac{\partial C_{0,1}}{\partial t}=\phi\left( 1-\alpha\right)\beta^{c}X_{0,1}-\left( \mu+\psi\gamma+\zeta+\xi_{i}\left( t \right) \right)C_{0,1}+\omega_{i}\left( t \right)C_{0,0}+\pi C_{0,3}$$

$$\frac{\partial X_{0,2}}{\partial t}=-\left( \mu+\gamma+\pi+\omega_{0}\left( t \right)+\chi\left( 1-\alpha\right)\beta^{c} \right)X_{0,2}+\xi_{i}\left( t \right)X_{0,0}+\zeta X_{0,3}$$

$$\frac{\partial C_{0,2}}{\partial t}=\chi\left( 1-\alpha\right)\beta^{c}X_{0,2}-\left( \mu+\gamma+\pi+\omega_{0}\left( t \right) \right)C_{0,2}+\xi_{i}\left( t \right)C_{0,0}+\zeta C_{0,3}$$

$$\frac{\partial X_{0,3}}{\partial t}=-\left( \mu+\psi\gamma+\zeta+\pi+\phi\chi\left( 1-\alpha\right)\beta^{c} \right)X_{0,3}+\omega_{0}\left( t \right)X_{0,2}+\xi_{0}\left( t \right)X_{0,1}$$

$$\frac{\partial C_{0,3}}{\partial t}=\phi\chi\left( 1-\alpha\right)\beta^{c}X_{0,3}-\left( \mu+\psi\gamma+\zeta+\pi\right)C_{0,3}+\omega_{0}\left( t \right)C_{0,2}+\xi_{0}\left( t \right)C_{0,1}$$

For incarcerated PWID:

$$\frac{\partial X_{1,0}}{\partial t}=p_{1}\theta(t)-\left( \mu+\tau+\omega_{1}\left( t \right)+\left( 1-\alpha\right)\beta^{p} \right)X_{1,0}+\zeta X_{1,1}+\gamma X_{0,0}+\delta{(X}_{2,0}+X_{3,0})+{\gamma X}_{0,2}+\delta(X_{2,2}+X_{3,2})+(1-c)\psi\gamma( X_{0,1}+X_{0,3}) +(1-c) \psi\delta(X_{2,1}+ X_{2,3}+ X_{3,1}+ {\psi X}_{3,3})$$

$$\frac{\partial C_{1,0}}{\partial t}=\left( 1-\alpha\right)\beta^{p}X_{1,0}-\left( \mu+\tau+\omega_{1}\left( t \right) \right)C_{1,0}+\zeta C_{1,1}+\gamma C_{0,0}+ \delta{(C}_{2,0}+C_{3,0})+(1-c)\psi\gamma( C_{0,1}+C_{0,3})+(1-c)\psi\delta(C_{2,1}+ C_{2,3}+ C_{3,1}+ C_{3,3})+{\gamma C}_{0,2}+\delta(C_{2,2}+C_{3,2})$$

$$\frac{\partial X_{1,1}}{\partial t}=-\left( \mu+\tau+\zeta+\phi\left( 1-\alpha\right)\beta^{p} \right)X_{1,1}+\omega_{1}\left( t \right)X_{1,0}+c\psi\gamma{(X}_{0,1}+X_{0,3})+c\psi\delta\left( X_{2,1}+X_{3,1}+X_{2,3}+X_{3,3} \right)$$

$$\frac{\partial C_{1,1}}{\partial t}=\phi\left( 1-\alpha\right)\beta^{p}X_{1,1}-\left( \mu+\tau+\zeta\right)C_{1,1}+\omega_{1}\left( t \right)C_{1,0}+c\psi\gamma(C_{0,1}+C_{0,3})+c\psi\delta(C_{2,1}+C_{3,1}+C_{2,3}+C_{3,3})$$

$$\frac{\partial X_{1,2}}{\partial t}=\frac{\partial C_{1,2}}{\partial t}=\frac{\partial X_{1,3}}{\partial t}=\frac{\partial C_{1,3}}{\partial t}=0$$

For recently released PWID:

$$\frac{\partial X_{2,0}}{\partial t}=p_{2}\theta\left( t \right)-\left( \mu+\delta+2+\omega_{2}\left( t \right)+\xi_{2}\left( t \right)+\eta\left( 1-\alpha\right)\beta^{c} \right)X_{2,0}+\zeta X_{2,1}+\pi X_{2,2}+\tau X_{1,0}+\left( 1-d \right)\tau X_{1,1}$$

$$\frac{\partial C_{2,0}}{\partial t}=\eta\left( 1-\alpha\right)\beta^{c}X_{2,0}-\left( \mu+\delta+2+\omega_{2}\left( t \right)+\xi_{2}\left( t \right) \right)C_{2,0}+\zeta C_{2,1}+\pi C_{2,2}+\tau C_{1,0}+\left( 1-d \right)\tau C_{1,1}$$

$$\frac{\partial X_{2,1}}{\partial t}=-\left( \mu+\psi\delta+2+\zeta+\xi_{2}\left( t \right)+\eta\phi\left( 1-\alpha\right)\beta^{c} \right)X_{2,1}+\omega_{i}\left( t \right)X_{2,0}+\pi X_{2,3}+d\tau X_{1,1}$$

$$\frac{\partial C_{2,1}}{\partial t}=\eta\phi\left( 1-\alpha\right)\beta^{c}X_{2,1}-\left( \mu+\psi\delta+2+\zeta+\xi_{2}\left( t \right) \right)C_{2,1}+\omega_{i}\left( t \right)C_{2,0}+\pi C_{2,3}+d\tau C_{1,1}$$

$$\frac{\partial X_{2,2}}{\partial t}=-\left( \mu+\delta+2+\pi+\omega_{2}\left( t \right)+\eta\chi\left( 1-\alpha\right)\beta^{c} \right)X_{2,2}+\xi_{i}\left( t \right)X_{2,0}+\zeta X_{2,3}$$

$$\frac{\partial C_{2,2}}{\partial t}=\eta\chi\left( 1-\alpha\right)\beta^{c}X_{2,2}-\left( \mu+\delta+2+\pi+\omega_{2}\left( t \right) \right)C_{2,2}+\xi_{i}\left( t \right)C_{2,0}+\zeta C_{2,3}$$

$$\frac{\partial X_{2,3}}{\partial t}=-\left( \mu+\psi\delta+2+\zeta+\pi+\eta\phi\chi\left( 1-\alpha\right)\beta^{c} \right)X_{2,3}+\omega_{2}\left( t \right)X_{2,2}+\xi_{2}\left( t \right)X_{2,1}$$

$$\frac{\partial C_{2,3}}{\partial t}=\eta\phi\chi\left( 1-\alpha\right)\beta^{c}X_{2,3}-\left( \mu+\psi\delta+2+\zeta+\pi\right)C_{2,3}+\omega_{2}\left( t \right)C_{2,2}+\xi_{2}\left( t \right)C_{2,1}$$

For previously incarcerated PWID:

$$\frac{\partial X_{3,0}}{\partial t}=p_{3}\theta\left( t \right)-\left( \mu+\delta+\omega_{3}\left( t \right)+\xi_{3}\left( t \right)+\left( 1-\alpha\right)\beta^{c} \right)X_{3,0}+\zeta X_{3,1}+\pi X_{3,2}+2X_{2,0}$$

$$\frac{\partial C_{3,0}}{\partial t}=\left( 1-\alpha\right)\beta^{c}X_{3,0}-\left( \mu+\delta+\omega_{3}\left( t \right)+\xi_{3}\left( t \right) \right)C_{3,0}+\zeta C_{3,1}+\pi C_{3,2}+2C_{2,0}$$

$$\frac{\partial X_{3,1}}{\partial t}=-\left( \mu+\psi\delta+\zeta+\xi_{3}\left( t \right)+\phi\left( 1-\alpha\right)\beta^{c} \right)X_{3,1}+\omega_{i}\left( t \right)X_{3,0}+\pi X_{3,3}+2X_{2,1}$$

$$\frac{\partial C_{3,1}}{\partial t}=\phi\left( 1-\alpha\right)\beta^{c}X_{3,1}-\left( \mu+\psi\delta+\zeta+\xi_{3}\left( t \right) \right)C_{3,1}+\omega_{i}\left( t \right)C_{3,0}+\pi C_{3,3}+2C_{2,1}$$

$$\frac{\partial X_{3,2}}{\partial t}=-\left( \mu+\delta+\pi+\omega_{3}\left( t \right)+\chi\left( 1-\alpha\right)\beta^{c} \right)X_{3,2}+\xi_{3}\left( t \right)X_{3,0}+\zeta X_{3,3}+2X_{2,2}$$

$$\frac{\partial C_{3,2}}{\partial t}=\chi\left( 1-\alpha\right)\beta^{c}X_{3,2}-\left( \mu+\delta+\pi+\omega_{3}\left( t \right) \right)C_{3,2}+\xi_{3}\left( t \right)C_{3,0}+\zeta C_{3,3}+2C_{2,2}$$

$$\frac{\partial X_{3,3}}{\partial t}=-\left( \mu+\psi\delta+\zeta+\pi+\phi\chi\left( 1-\alpha\right)\beta^{c} \right)X_{3,3}+\omega_{i}\left( t \right)X_{3,2}+\xi_{i}\left( t \right)X_{3,1}+2X_{2,3}$$

$$\frac{\partial C_{3,3}}{\partial t}=\phi\chi\left( 1-\alpha\right)\beta^{c}X_{3,3}-\left( \mu+\psi\delta+\zeta+\pi\right)C_{3,3}+\omega_{i}\left( t \right)C_{3,2}+\xi_{i}\left( t \right)C_{3,1}+2C_{2,3}$$

With forces of infection:

$$\beta^{c}=\lambda\frac{\bar{C}_{0}+\eta\bar{C}_{2}+\bar{C}_{3}}{\bar{X}_{0}+\eta X_{2}+X_{3}+\bar{C}_{0}+\eta\bar{C}_{2}+\bar{C}_{3}}$$

$$\beta^{p}=\eta\lambda\frac{\bar{C}_{1}}{{X_{1}+\bar{C}}_{1}}$$

where, $\bar{X}_{i}=X_{i,0}+{\phi X}_{i,1}+{\chi X}_{i,2}+{\phi\chi X}_{i,3}$

$$\bar{C}_{i}=C_{i,0}+{\phi C}_{i,1}+{\chi C}_{i,2}+{\phi\chi C}_{i,3}$$

## Parameterising and calibrating the incarceration sub-model

We created an incarceration sub-model by adapting the incarceration component of the full model. This adapted incarceration model (see Supplementary Fig. S1) stratified PWID by incarceration status (never incarcerated, currently incarcerated or previously incarcerated), and where PWID initiated injecting (in the community having never been incarcerated (k=1); in prison (k=2); or in the community having previously been incarcerated(k=3)). PWID are incarcerated and re-incarcerated with rates $\gamma$ and $\delta$, respectively, and PWID are released from prison at a fixed rate $\tau$. The adapted incarceration model was calibrated to data from the Social Networks Among Appalachian People study (denoted as ‘SNAP’) on the proportion of community PWID with a history of incarceration and their mean number of incarcerations for different durations of injecting (data used is in Supplementary Table S1). Using the adapted model, 1,000 PWID were followed throughout their injecting career for 18 years from the onset of injecting, with a proportion $p_{k}^{*}$ of the 1,000 PWID starting in each compartment with on average $m$ prior incarcerations among those starting injecting in prison or in the community having been previously incarcerated.

**Supplementary Fig. S1**: Model schematics of the incarceration sub model.

**Supplementary Table S1:** Data used to calibrate the incarceration sub-model.

| **Duration of injecting** | **Proportion ever incarcerated** | **Mean number of times incarcerated (of those ever incarcerated)** |
| --- | --- | --- |
| <3 Years | 60.0%  (95% CI: 43.3 – 75.1) | 3.2  (95% CI: 2.2 – 4.2) |
| 3-6 Years | 91.3%  (95% CI: 79.2 – 97.6) | 6.1  (95% CI: 3.8 – 8.4) |
| 6-9 Years | 92.3%  (95% CI: 81.4 – 97.9) | 6.8  (95% CI: 4.7 – 8.8) |
| 9-12 Years | 78.4%  (95% CI: 64.7 – 88.7) | 11.2  (95% CI: 3.3 – 19.0) |
| 12-15 Years | 94.4%  (95% CI:81.3 – 99.3) | 15.2  (95% CI: 6.2 – 24.3) |
| 15-18 Years | 95.7%  (95% CI: 78.1 – 99.9) | 14.0  (95% CI: 5.0 – 22.9) |

An ABC SMC routine(1) is used to obtain a sample of 5,000 incarceration-related parameter sets that fit the incarceration data. The data, (Table S1), consists of data points, denoted by $\left\{ x_{d}^{a} \right\}$, which are the proportions of PWID in the community that have ever been incarcerated ($a=0$) and the mean number of times previously incarcerated community PWID have been incarcerated ($a=1$), by duration injecting, d. The distance function was taken to be the sum of the normalised square errors, i.e. $\sum_{d} \sum_{a=0,1} \frac{{(y_{d}^{a}-x_{d}^{a})}^{2}}{x_{d}^{a}}$, where the simulated data is denoted by $\left\{ y_{d}^{a} \right\}$. The distance function was used because of the differences in the order of magnitude between the proportion of PWID that have ever been incarcerated and their mean number of incarcerations.

At the first iteration of the ABC SMC, parameters were sampled from their prior distributions (Table 1 in main paper). At subsequent iterations, the parameter sets from the previous iteration were sampled from with weights dependent upon the prior likelihood of the parameter set and the perturbation kernel (uniform in this implementation). The sampled parameter sets were perturbed using a uniform perturbation kernel, which could perturbate each parameter by at most +/- 5% of the prior range, so as to still be within the prior ranges, accepting those that gave model fits whose error, measured by the distance function, was less than that iteration’s tolerance. At each iteration of the ABC SMC, the tolerance was set to be the 75th percentile of the output of the distance function of accepted model fits in the previous iteration. The adaptive tolerances guaranteed a monotonically decreasing sequence of tolerances. The ABC SMC routine was stopped when the tolerances began to stabilise (<1% relative difference between successive iterations). The 5,000 parameter sets obtained at the end of the routine were then used directly to parameterise the transmission dynamics of the full model.

## Parameterising and calibrating the full model

### Calibrating the increase in injecting initiation

Based on baseline SNAP data on the number of named social network members that were injectors (analysis for this project, Jennifer Havens and April Young), we assumed a PWID population size of 700 in Perry County for 2009. The 503 participants in the SNAP cohort named 2634 alters (including drug, sex, and social support partners) of whom 897 were determined to be other study participants(2). Analysis was completed on non-study participants to determine if (a) they injected drugs and (b) to de-duplicate the list of non-study alters. This analysis resulted in the estimated PWID population size of 700. Uncertainty was associated around this parameter (+/-20%). In addition, consistent with data from other sources(3), baseline SNAP data on when PWID started injecting also suggested the recruitment of new PWID increased dramatically between 1990 and 2000. This is shown in Supplementary Fig. S2, which shows an 8-fold greater number of PWID in SNAP reported that they started injecting in 2000 compared to 1990. However, it is not possible to ascertain whether this increase is solely due to greater recruitment of PWID, or alternatively that there was a smaller increase in recruitment paired with some PWID from that time already ceasing injecting. Therefore, the modelled increase in the initiation of injecting differs based on sampled values of cessation and mortality rates, with greater increases in the initiation of injecting occurring with lower cessation and mortality rates.

We assume that the number of PWID who initiated injecting increased linearly from $\theta\left( t \right)=\theta_{0}/\sigma$ in 1990 up to $\theta\left( t \right)=\theta_{0}$ in 2000, where $\sigma\geq1$. Therefore, $\theta\left( t \right)$ is given by

$\theta\left( t \right)= \left\{ \begin{aligned} \theta_{0}/\sigma\\ \frac{\theta_{0}}{\sigma}+\frac{(t-1990){(1-\frac{1}{\sigma})\theta}_{0}}{10} \\ \theta_{0} \end{aligned} \right.$     $\begin{matrix} if t<1990 \\ if 1990\leq t<2000 \\ if t\geq2000 \end{matrix}$

We sampled from a range of mortality rates and cessation rates (Table 2 in the main paper) and then calibrated the increase in initiating of injecting so that in 2009 there would be eight times more current PWID that started injecting in 2000 compared to current PWID that started injecting in 1990. We simultaneously calibrated the rate that PWID start injecting post-2000 ($\theta_{0}$) such that the model achieves the sampled PWID population size from SNAP data in 2009.

**Supplementary Fig. S2:** Frequency of PWID by the year of first injection from the SNAP study, grouped by 5-year intervals.

### Parameterising the effect of OST on incarceration rates

A study of a cohort of male heroin users (with a history of heroin injection) recruited in prisons in New South Wales, Australia, found that whilst OST at prison release was not associated with reduced rates of re-incarceration, the average risk of being re-incarcerated was 20% lower (AHR: 0.80, 95% CI: 0.71-0.90) whilst participants were retained in post-release treatment(4). Similarly, a study of a cohort of PWID in Vancouver (Vancouver Injecting Drug Users Study - VIDUS) found that being on OST was associated with a 34% (AOR: 0.66, 95% CI: 0.58-0.76) reduction in the odds of being incarcerated in the previous 6 months(5). Due to the temporal differences in the measurement of OST exposure (at interview) and incarceration outcome (in the last 6 months), this association could have been explained by the limited access to OST within prisons. However, analyses of PWID who enrolled in OST during the study found that a significantly higher proportion of these PWID reported recent incarceration prior to OST enrolment (37.7%) than after initiating OST (31.8%), suggesting that OST reduced incarceration rates. To account for the wide range in the estimates of the effect of OST on reducing rates of incarceration, this parameter value was sampled from a uniform distribution with range (0.58-0.90).

### Parameterising NSP loss to follow-up rates

A simple model of NSP enrolment and loss to follow-up was developed (Supplementary Fig. S3) which stratified PWID by their NSP status; whether they had not used NSP in the last 12 months, whether they were current NSP users, or whether they were not current users of NSP but had been a NSP user in the last 12 months. PWID not currently using NSP start using NSP at a constant rate, $\alpha$, whilst those currently using NSP stop using NSP at a constant rate, $\beta$.

This simple model was initiated with a population size of 1000 PWID, which was randomly distributed amongst the three NSP statuses, and was then run to equilibrium ($t_{0}$). The model was then run for a year ($t_{1}$), tracking:

- $p_{1}$, the proportion of recent NSP users (currently or in the last 12 months) at $t_{0}$ who were recent NSP users at $t_{1}$.
- $p_{2}$, the proportion of non-recent NSP users (not in the last 12 months) at $t_{0}$ who were recent NSP users (currently or in the last 12 months) at $t_{1}$.

**Supplementary Fig. S3:** Model schematic of simplified model of NSP recruitment and loss to follow-up.

The NSP recruitment and loss to follow-up rates ($\alpha$ and $\beta$, respectively) were then calibrated to give values of $p_{1}$ and $p_{2}$ which matched the transition probabilities found in the cohort study(6). This was repeated 1,000 times, each time with $p_{1}$ being calibrated to 68.6%, corresponding to the probability of a “Direct NSP user” (one who has obtained syringes from a NSP in the last 12 months) maintaining their status after a year, and $p_{2}$ being calibrated to a value sampled uniformly from the range (32.8 - 37.5%), with the maximum and minimum of the range corresponding to the probabilities of an “Indirect NSP user” (one who obtains syringes or other injecting equipment from a NSP user but not directly from a NSP) or an “Isolated PWID” (one who does not obtain syringes directly or indirectly from a NSP) transitioning to a “Direct NSP user” after a year, respectively. This gave a distribution of 1,000 NSP loss to follow-up rates in the range 1.12-1.32, corresponding to an average 9.1-10.7 months on NSP, which were then sampled from to parameterise the NSP loss to follow-up rates in the final model.

## Uncertainty Analysis

**Supplementary Fig. S4**: Results of the ANCOVA analysis detailing which model parameters’ uncertainty contributes most to the variability in the impact (% of HCV infections averted) of the comprehensive harm reduction scenario S4. The figure plots the proportion of the model outcome’s sum-of-squares contributed by each parameter. Parameters contributing less than 1.0% of uncertainty are not shown.

**References**

1. Toni T, Welch D, Strelkowa N, Ipsen A, Stumpf MP. Approximate Bayesian computation scheme for parameter inference and model selection in dynamical systems. J R Soc Interface. 2009;6(31):187-202.

2. Young AM, Rudolph AE, Su AE, King L, Jent S, Havens JR. Accuracy of name and age data provided about network members in a social network study of people who use drugs: implications for constructing sociometric networks. Annals of epidemiology. 2016;26(11):802-9.

3. Zibbell JE, Iqbal K, Patel RC, Suryaprasad A, Sanders KJ, Moore-Moravian L, et al. Increases in hepatitis C virus infection related to injection drug use among persons aged≤ 30 years-Kentucky, Tennessee, Virginia, and West Virginia, 2006-2012. MMWR Morbidity and mortality weekly report. 2015;64(17):453-8.

4. Larney S, Toson B, Burns L, Dolan K. Effect of prison-based opioid substitution treatment and post-release retention in treatment on risk of re-incarceration. Addiction. 2012;107(2):372-80.

5. Werb D, Kerr T, Marsh D, Li K, Montaner J, Wood E. Effect of methadone treatment on incarceration rates among injection drug users. Eur Addict Res. 2008;14(3):143-9.

6. Green TC, Bluthenthal RN, Singer M, Beletsky L, Grau LE, Marshall P, et al. Prevalence and predictors of transitions to and away from syringe exchange use over time in 3 US cities with varied syringe dispensing policies. Drug Alcohol Depend. 2010;111(1-2):74-81.
